# Downregulated exosomal microRNA-148b-3p in cancer associated fibroblasts enhance chemosensitivity of bladder cancer cells by downregulating the Wnt/β-catenin pathway and upregulating PTEN

**DOI:** 10.1007/s13402-020-00500-0

**Published:** 2021-01-10

**Authors:** Guang Shan, Xike Zhou, Juan Gu, Daoping Zhou, Wei Cheng, Huaiguo Wu, Yueping Wang, Tian Tang, Xuedong Wang

**Affiliations:** 1 Department of Urology, RenMin Hospital of Wuhan University, Wuhan, 430060 Hubei People’s Republic of China; 2 Department of Medical Laboratory Science, The Fifth People’s Hospital of Wuxi, Nanjing Medical University, 1215 Guangrui Road, Jiangsu 214000 Wuxi, People’s Republic of China; 3 Department of Pathology, The Fifth People’s Hospital of Wuxi, The Medical School of Jiangnan University, Jiangsu 214000 Wuxi, People’s Republic of China; 4 Center for Precision Medicine, Anhui No.2 Provincial People’s Hospital, Hefei 230041 Anhui, People’s Republic of China; 5 Department of Biology, College of Arts & Science, Massachusetts University, MA 02125 Boston, USA; 6 Department of Oncology, RenMin Hospital of Wuhan University, Hubei 430060 Wuhan, People’s Republic of China

**Keywords:** Bladder cancer, Cancer-associated fibroblast, Exosome, microRNA-148b-3p, PTEN, Epithelial-mesenchymal transition

## Abstract

**Objective:**

Exosomes derived from cancer-associated fibroblasts (CAFs) are known as important drivers of tumor progression. Previously, microRNA (miR)-148b-3p has been found to be upregulated in bladder cancers as well as in body fluids (blood, urine) of bladder cancer patients. Here, we aimed to explore the role of CAF-derived exosome miR-148b-3p in bladder cancer progression and chemosensitivity.

**Methods:**

Transwell, MTT, flow cytometry and colony formation assays were applied to assess the effects of CAF-derived exosomes on bladder cancer cell metastasis, epithelial-mesenchymal transition (EMT) and chemosensitivity. A dual luciferase reporter assay was employed to evaluate the targeting relationship between miR-148b-3p and PTEN. Gain- and loss- of function assays were conducted to explore the roles of miR-148b-3p and PTEN in the behavior of bladder cancer cells. The role of PTEN in the metastasis, EMT and chemosensitivity of bladder cancer cells was assessed both *in vivo* and *in vitro*.

**Results:**

We found that CAF-derived exosomes promoted the metastasis, EMT and drug resistance of bladder cancer cells. We also found that CAF-derived exosomes could directly transport miR-148b-3p into bladder cancer cells. In a xenograft mouse model we found that CAF-derived exosomes increased miR-148b-3p expression levels and promoted tumor proliferation, metastasis and drug resistance. PTEN was validated as a target of miR-148b-3p. Concordantly, we found that PTEN overexpression inhibited EMT, metastasis and chemoresistance in bladder cancer cells, reversing the tumor promoting effects of miR-148b-3p via the Wnt/β-catenin pathway.

**Conclusions:**

Our results suggest that miR-148b-3p downregulation in CAF-derived exosomes, thereby inhibiting the Wnt/β-catenin pathway and promoting PTEN expression, may offer potential opportunities for bladder cancer treatment.

**Electronic supplementary material:**

The online version of this article (10.1007/s13402-020-00500-0) contains supplementary material, which is available to authorized users.

## 1 Introduction

Bladder cancer is the most common cancer of the genitourinary tract [1]. Tobacco smoking, genetic factors and exposure to aromatic amines and other industrial chemicals are considered to contribute to bladder cancer development [2]. Nearly 25% of the patients are initially diagnosed at metastatic stages, and 50% of those diagnosed at early stages will eventually progress to metastatic disease [3]. The treatment options largely depend on the early diagnosis and usually include transurethral resection followed by partial or radical cystectomy, intravesical immunotherapy, chemotherapy or radiotherapy [4]. Unfortunately, the therapeutic effects of advanced bladder cancers are still limited, as indicated by a 5-year survival rate of 35% for patients diagnosed at regional stages and 5% for patients diagnosed at distant stages [5]. Drug resistance and recurrence are significant barriers to an effective treatment for bladder cancer [6]. Therefore, it is crucial to identify strategies that may reverse drug resistance to improve the management of this disease.

Cancer-associated fibroblasts (CAFs) are important components of the tumor microenvironment (TME) that interact with cancer cells to promote tumor development and progression [7, 8]. Exosomes secreted from CAFs are believed to be important contributors to tumor development [9, 10]. Exosomes may carry a range of bioactive molecules, such as signaling peptides, microRNAs (miRs) and DNA, among which miRs can be stably transferred and recognized to regulate cell-to-cell communication and to exert essential functions in the coupling systems between cancer cells and the TME [11]. The miR-148/152 family, including miR-148a, miR-148b and miR-152, contributes to the development of various tumors. The expression of miR-148b-3p has been found to be upregulated in bladder cancer [12] and expression of the miR-148/152 family has been found to enhance the chemosensitivity of breast cancer cells to doxorubicin (DOX) [13]. There is a growing body of evidence underscoring the value of miR-148b detection in body fluids (blood and urine) of bladder cancer and breast cancer patients [14, 15]. Importantly, Kabir et al. found that miR-148b may be elevated in senescent fibroblasts and target the phosphatase and tensin homologue deleted on chromosome 10 (PTEN) [16]. Indeed, a previous study indicated that low PTEN levels correlate with an unfavorable prognosis in human bladder cancer [17]. Interestingly, Ahmad et al.. revealed that a combination of β-catenin activation and PTEN loss contributes to bladder cancer initiation [18], whereas Yoshida et al.. emphasized that the Wnt/β-catenin pathway may be involved in bladder cancer development [19]. Thus, it is reasonable to hypothesize that a regulatory network may exist involving miR-148b-3p, PTEN and the Wnt/β-catenin pathway that underlies the development and clinical behavior of bladder cancer.

## Materials and methods

### Ethics statement

This study was approved and supervised by the ethics committee of the Renmin Hospital of Wuhan University. All subjects signed informed consent forms. All animal experiments performed in this study are in conformity with the management of local laboratory animals and the medical ethics committee of the Renmin Hospital of Wuhan University. Significant efforts were made to minimize both the number of animals used and their suffering.

### Cell culture

Bladder cancer tissue specimens and corresponding normal bladder tissue specimens were collected from 60 patients (Renmin Hospital of Wuhan University), none of whom received chemotherapy or radiotherapy before surgery. The fresh tissue specimens were sliced into small sections and subsequently disaggregated in 160 µg/ml collagenase A and 25 µg/ml hyaluronidase (both from Sigma-Aldrich, Merck KGaA, Darmstadt, Germany) at 37 °C for 2 h. Next, cells were collected and cultured in Dulbecco’s Modified Eagle’s Medium (DMEM)/F12 containing 100 U/ml penicillin, 100 µg/ml streptomycin and 10% fetal bovine serum (FBS) (Invitrogen Inc., Carlsbad, ca., USA) without exosomes (centrifuged at 200,000 g for 18 h for exosome depletion). After 2 ~ 3 passages, uniform cancer-associated fibroblasts (CAFs) were obtained. All CAFs used in the experiments were cultured for less than 10 passages. Human bladder cancer-derived cells 5637 and T24 (American Type Culture Collection, Manassas, VA, USA) were cultured in Roswell Park Memorial Institute (RPMI)-1640 medium supplemented with 10% FBS (without exosomes), 100 U/ml penicillin and 100 µg/ml streptomycin at 37 °C in a humidified atmosphere with 5% CO_2_.

### Preparation of conditioned medium (CM)

CAFs and normal fibroblasts (NFs) were cultured in DMEM/F12 supplemented with 100 U/ml penicillin, 100 µg/ml streptomycin and 10% exosome-free FBS. Supernatants were collected by 700 g centrifugation for 10 min and referred to as conditioned media (CAF-CM and NF-CM).

### Immunocytochemistry

Cleaned, acidified and sterilized coverslips were put into a cell culture dishes after which cells were cultured on top of them for 24 ~ 48 h. After the coverslips were covered with cells, the culture medium was removed and the cells were washed with PBS, fixed in 4% formaldehyde for 15 min and incubated in 0.3% Triton X-100 for 20 min. After a subsequent wash with PBS, the cells were incubated in 1% bovine serum albumin (BSA) for 30 min and subsequently with a primary antibody at 37 °C for 1 h. Next, the cells were washed again with PBS and incubated with a secondary antibody in a wet box for 30 min. After being washed with PBS, the cells were stained with 2,4-diaminobutyric acid, counterstained with hematoxylin, dehydrated, cleared and sealed for observation. The antibodies directed against vimentin (1:100, ab8978), keratin (1:20, ab8068), fibroblast activation protein (FAP, 1:50, ab28244) and alpha smooth muscle actin (α-SMA, 1:20, ab32575) were all purchased from Abcam Inc. (Cambridge, MA, USA).

### Isolation of exosomes and transmission electron microscopy (TEM)

Exosomes were collected from CAF-CM and NF-CM by differential ultracentrifugation. After washing with PBS, the exosomes were prefixed with 2.5% glutaraldehyde in PBS (pH 7.4) for 2 h and fixed again with 1% osmium tetroxide in PBS for another 2 h. Next, the exosomes were incubated on a glow-discharged copper grid for 1 min and stained with a drop of 2% phosphotungstic acid aqueous solution, after which excess buffer was carefully drained from the edge of the copper grid with filter papers. Next, the mesh was dyed with 2% uranyl acetate (pH 7.0) for 40 s, after which the samples were air-dried at room temperature and examined by TEM at 80 keV.

### Flow cytometry

A total of 1 × 10^6^ exosomes were collected, resuspended in PBS and added to 2 µl fluorescent antibodies and homotypic controls of the same volume, followed by a 30 min incubation on ice. After washing with fluorescence-activated cell sorting buffer and fixing with 10% formalin, the positive rate of antigens was measured using a flow cytometer (MoFlo Astrios EQ, Beckman Coulter, Inc., ca., USA). The antibodies used were anti-CD63 (1:1000, ab59479), anti-CD81 (1:1000, ab79559) and anti-tumor susceptibility gene 101 (TSG101, 1:1000, ab30871) (all purchased from Abcam).

### Western blot analysis

Total protein from each sample was extracted, after which the concentration was determined according to the instructions of the bicinchoninic acid kit (Thermo Scientific Pierce, Rockford, IL, USA). Next, the extracted proteins were boiled at 95 °C for 10 min, after which 30 µg of each sample was separated by 10% w/v sodium dodecyl sulfate polyacrylamide gel electrophoresis from 80 to 120 V. Next, the proteins were transferred to polyvinylidene fluoride membranes using a wet transfer method at 100 mV for 45–70 min. Subsequently, the membranes were blocked in 5% BSA at room temperature for 1 h and incubated with primary antibodies directed against vimentin (1:1000, ab8978), N-cadherin (1:1000, ab76057), E-cadherin (1:500, ab15148), Bax (1:1000, ab32503), caspase-3 (1:500. ab13847), β-catenin (1:5000, ab32572), Wnt1 (1:100, ab15251), c-Myc (1:1000, ab32072), cytochrome c (1:5000, ab133504), caspase-9 (1:2000, ab202068) and PTEN (1:5000, ab32199) (all from Abcam) at 4 °C overnight. Next, the membranes were rinsed in Tris-buffered saline with Tween (TBST) 3 times (10 min/time) and incubated with a secondary antibody labeled with horseradish peroxidase at room temperature for 1 h. After TBST washing, the proteins were visualized using an enhanced chemiluminescence reagent and developed using a Gel EZ imager (Bio-Rad Laboratories, Hercules, ca., USA). Finally, the target bands were analyzed using ImageJ software (National Institutes of Health, Bethesda, Maryland, USA).

### Nanoparticle tracking analysis

Exosomes were diluted in 1 ml tris(pyrazolyl)methane for further analysis. Next, ZetaView PMX 110 (Particle Metrix GmbH, Microtrac, Meerbusch, Germany) was applied to measure the size and concentration of exosomes using the NanoSight Tracking Analysis tool.

### Immunofluorescence staining

The extracted exosomes were resuspended in 100 µl PKH67 working solution (Sigma-Aldrich), after which the cells were adjusted to 10^7^ cells/ml and incubated at 2 ~ 8 °C for 15–30 min. Following centrifugation and supernatant removal, the cells were washed in PBS and resuspended. A 500 µl suspension was used to detect cell apoptosis at an excitation wavelength of 490 nm and an emission wavelength of 502 nm on a flow cytometer. Exosome internalization was observed under a laser scanning confocal microscope after co-cultivation of stained exosomes with cells.

### 3-(4,5-dimethylthiazol-2-yl)-2,5-diphenyltetrazolium bromide (MTT) viability assay

A single cell suspension was prepared at 1 × 10^5^ cells/ml using cells in the exponential growth phase treated with different concentrations of paclitaxel (PTX, 0.05, 0.10, 0.15, 0.30, 0.50, 0.80 and 1.00 µM) or DOX (0.5, 1.0, 1.5, 2.0, 2.5, 3.0 and 3.5 µM), after which the cells were inoculated into 96-well plates at a density of 200 µl/well. After culturing for 48 h, cell viability was detected using 20 µl MTT solution (5 mg/ml, Sigma-Aldrich). Next, 150 µl dimethyl sulfoxide was added to each well for 15 min to fully dissolve the crystals. The absorbance (A) at a wavelength of 490 nm was measured in each well using a microplate reader. The cell proliferation rate was expressed as (A490 in the experimental group – A490 in the blank group)/(A490 in the negative control (NC) group – A490 in the blank group) × 100%. The IC50 (half maximal inhibitory concentration) value of each drug was determined by calculating the dose required for 50% cell viability.

### Transwell invasion assay

Bladder cancer 5637 and T24 cells were collected and added to serum-free culture medium containing 0.1% BSA. After resuspension at a density of 1 × 10^5^ cells/ml, the cells were placed into the apical Transwell chambers, while the basolateral chambers were supplemented with DMEM containing 10% PBS. Subsequently, theTranswell chambers were incubated at 37 °C in a 5% CO_2_ atmosphere for 24 h, after which the filter membranes were removed, rinsed with PBS, fixed with 0.5% glutaraldehyde and stained with a crystal violet staining solution followed by incubation at 37 °C with 5% CO_2_ for 24 h. For the analysis, five fields (200 x) were selected randomly under a bright field microscope.

### Colony formation assay

Cells were harvested and trypsinized to prepare single-cell suspensions for use. After counting the cells, the cell concentration was adjusted to 5 × 10^2^ cells/ml and seeded into a 6‐well plate at 2 ml/well. Three parallel wells were used for each group of cells. Next, the 6‐well plate was placed in a 37 °C, 5% CO_2_ incubator for routine culture. The medium was changed once every 2 days for 14 days. On the 14th day the medium was discarded, after which the cells were rinsed three times with physiological saline and fixed in 1 ml 4% paraformaldehyde for 30 min. Next, the paraformaldehyde solution was discarded, and to each well 1 ml 5% crystal purple dye was added for one hour. After removing the staining solution, masses of ≥ 50 cells were counted as individual colonies under a bright light microscope.

### Quantitative reverse transcription PCR (qRT-PCR)

Total RNA was extracted using a one-step Trizol (Invitrogen) method, after which the quality of the extracted RNA was confirmed using ultraviolet analysis and formaldehyde denaturation electrophoresis. Fluorescent qRT-PCR was performed according to the instructions of the qRT-PCR kit (Thermo Fisher Scientific, Shanghai, China) with U6 as internal reference for miRNAs. The PCR primers used were devised and synthesized by Shanghai Sangon Biotechnology Co., Ltd. (Shanghai, China) (listed in Table 1). After the PCR reaction the amplification and dissolution curves were confirmed, and the data were analyzed using the 2^−ΔΔCt^ method.

**Table 1:** Primer sequences of RT-qPCR

Gene	Primer sequence
miR-148b-3p	F: 5’-TCAGTGCATCACAGAACTTTGT‐3’
	R: 5’-ACAAAGTTCTGTGATGCACTGA‐3’
miR-3187-3p	F: 5’-TTGGCCATGGGGCTGCGCGG‐3’
	R: 5’-CCGCGCAGCCCCATGGCCAA‐3’
miR-15b-5p	F: 5’-TAGCAGCACATCATGGTTTACA‐3’
	R: 5’-TGTAAACCATGATGTGCTGCTA‐3’
miR-27a-3p	F: 5’-TTCACAGTGGCTAAGTTCCGC‐3’
	R: 5’-GCGGAACTTAGCCACTGTGAA‐3’
miR-30a-5p	F: 5’-TGTAAACATCCTCGACTGGAAG‐3’
	R: 5’-CTTCCAGTCGAGGATGTTTACA‐3’
miR-152	F: 5’-TGTCCCCCCCGGCCCAGGTT‐3’
	R: 5’-GGTCCTTCCGGGCCCAAGTT‐3’
U6	F: 5’-CGCTTCGGCAGCACATATAC‐3’
	R: 5’-AATATGGAACGCTTCACGA‐3’

Note: RT-qPCR, reverse transcription quantitative polymerase chain reaction; miR-148b-3p, microRNA-148b-3p; miR-3187-3p,microRNA-3187-3p; miR-15b-5p,microRNA-15b-5p; miR-27a-3p,microRNA-27a-3p; miR-30a-5p,microRNA-30a-5p; miR-152,microRNA-152; F. forward; R, reverse

### Cell transfection and grouping

PTEN cDNA was cloned into a pcDNA3.1 vector (Invitrogen). In addition, miR-148b-3p mimic, miR-NC and miR-148b-3p inhibitor were designed and synthesized (Thermo Fisher, Shanghai, China). Lipofectamine 2000 (Invitrogen) was used for transfection as per manufacturer’s instructions.

NF-CM, CAF-CM, NF-exosomes and CAF-exosomes were incubated with 5637 and T24 cells and cultured with different concentrations of PTX and DOX for 48 h. Then, cell viability, invasion, flow cytometry and colony formation assays were carried out. After transfection of miR-148-3p inhibitor into CAFs, exosomes (CAF-exos/inhi) were collected. In addition, exosomes were co-incubated with 5637 or T24 cells and subsequently transfected with miR-148b-3p mimic (CAF-exos/inhi + mimic). Next, the cells were cultured with different concentrations of PTX and DOX for 48 h and subjected to cell viability, invasion, flow cytometry and colony formation assays. Additionally, miR-148b-3p mimic, miR-148b-3p inhibitor, miR-NC and anti-miR-NC were transfected into 5637 (5637-mimic, 5637-inhi, 5637-NC) and T24 (T24-mimic, T24-inhi, T24-NC) cells with upregulated or downregulated miR-148b-3p expression. Next, the cells were cultured with different concentrations of PTX and DOX for 48 h, after which cell viability, invasion, flow cytometry and colony formation assays were carried out. In addition, pcDNA3.1-PTEN was transfected into CAF-exo-treated 5637 and T24 cells.

### Fluorescein isothiocyanate (FITC)/propidium iodide (PI) staining

After 48 h of transfection, cells were washed in PBS and centrifuged at 2500 g, after which the supernatants were removed. Next, the cells were suspended in buffer and adjusted to a concentration of 1 × 10^6^ cells/ml. To 500 µl cell suspensions 5 µl annexin V-FITC and 10 µl PI (Invitrogen) were added, mixed and incubated at room temperature in the dark for 10 min, followed by detection on a flow cytometer.

### Dual luciferase reporter assay

A PTEN fragment containing the binding site of miR-148b-3p was cloned into a pmirGLO oligosaccharide enzyme vector (Promega, Madison, WI, USA), after which a pmirGLO-PTEN-wild type (Wt) reporting vector was constructed. A pmirGLO-PTEN-mutant type (Mut) reporting vector was constructed using a mutant binding site of miR-454-3p based on the pmirGLO-HOTAIR-Wt sequence. The constructed vectors were transfected into 5637 and T24 cells, which were subsequently transfected with miR-148-3p and miR-NC, respectively. After 48 h, luciferase activity was detected using a dual luciferase reporter assay system (Promega), and the relative activity was expressed as the ratio of firefly luciferase activity to Renilla luciferase activity.

### Dual luciferase reporter assay

Fifty-four pathogen-free BALB/c nude mice (4 ~ 6 weeks old, 20 ± 2 g) (Beijing Vital River Laboratory Animal Technology Co., Ltd, Beijing, China) were numbered based on body weight and randomly assigned into the 5637 group, the 5637 + CAF-exos group or the 5637 + CAF-exos/inhi group. Eighteen nude mice in each group were subcutaneously injected with 5637 cells (1 × 10^7^/0.2 ml/mouse). Next, the mice were injected with PTX (10 mg/kg, n = 06), DOX (3 mg/kg, n = 06) and PBS (n = 06) [20, 21] every three days via the tail vein. The mice in the 5637 + CAF-exos group were additionally injected with CAF-exos (2–3 µg/mouse), while those in the 5637 + CAF-exos/inhi group were injected with CAF-exos/inhi (2–3 µg/mouse). The tumor size (length × width) was measured every 3 days. After 21 days, the mice were euthanized using pentobarbital [22, 23], after which the tumors were removed. Nine tumors (3 with PTX treatment, 3 with DOX treatment and 3 with PBS treatment) were paraffin-embedded and sliced. The remaining tumors were ground into homogenates for protein and miR-148-3p expression detection.

### Immunohistochemistry

Tumor sections were incubated with an anti-Ki-67 antibody (1:500, ab15580, Abcam), after which the percentage of Ki-67-positive cells was quantified using Image-ProPlus 6.0.

### 4’,6-diamidino-2-phenylindole (DAPI) staining

DAPI solution was added to the cell culture medium (1/10 volume), after which the cells were cultured at 37 °C for 15 min, washed with PBS and evaluated under a fluorescence microscope.

### Statistical analysis

SPSS 21.0 (IBM Corp., Armonk, NY, USA) was used for data analysis. The Kolmogorov-Smirnov test was used to assess whether the data were normally distributed. The results are presented as mean ± standard deviation. Comparisons between two groups were analyzed using *t* test. Comparisons among multiple groups were analyzed using one-way analysis of variance (ANOVA) or two-way ANOVA, and pairwise comparisons after ANOVA were conducted by Tukey’s multiple comparisons test. *P* values were obtained using a two-tailed test, and *p* < 0.05 indicated a significant difference.

## 3 esults

### CAF-derived exosomes enhance metastasis and chemoresistance of bladder cancer cells

**Fig. 1:**
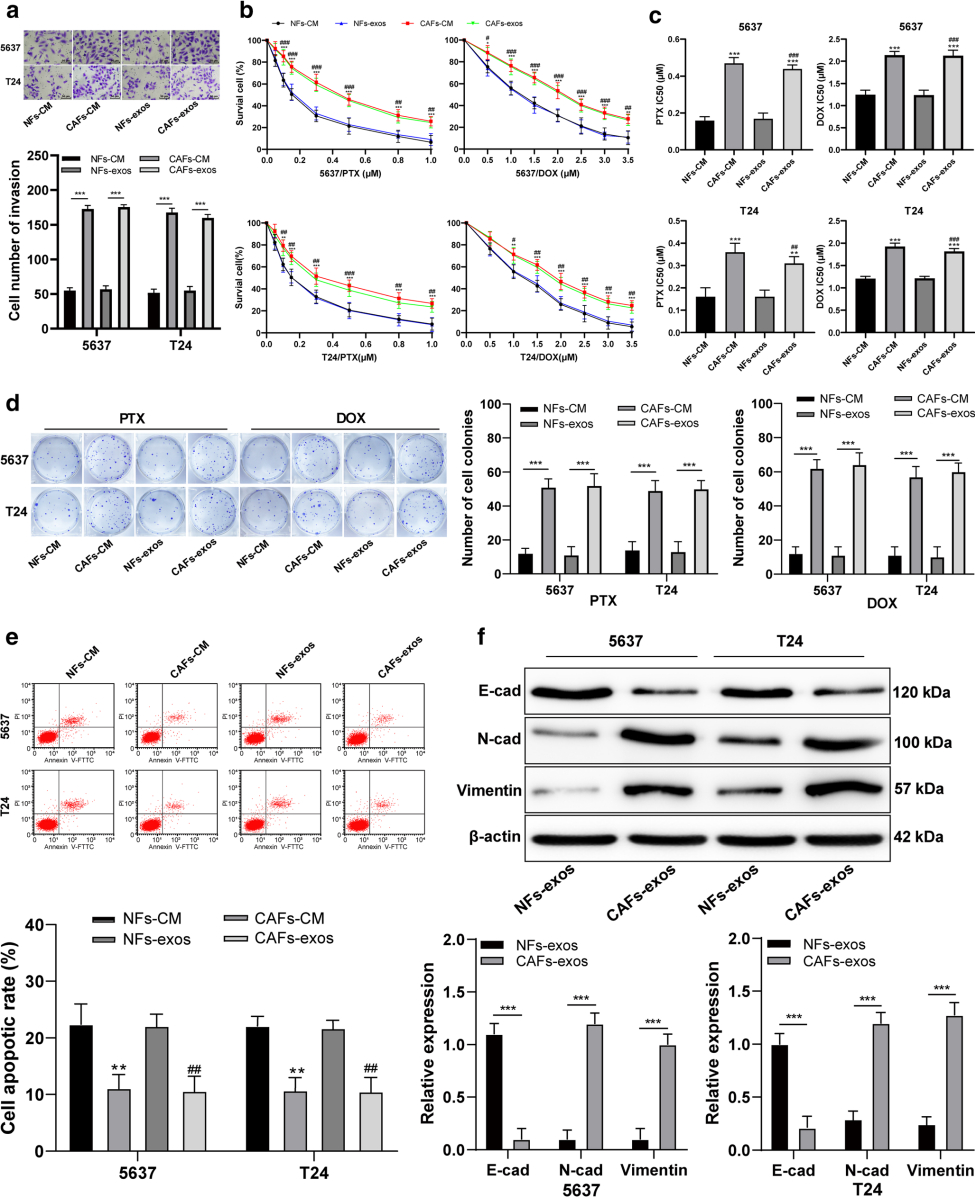
Exosomes secreted by CAFs promote the metastasis and chemoresistance of bladder cancer cells by accelerating EMT. **a**. Representative images and histogram of invaded cells treated with NF-CM or NF-exos, CAF-CM or CAF-exos, detected by Transwell assay, *** *p* < 0.001. **b**. Relative cell survival of 5637 and T24 cells (pretreatment with PTX and DOX for 48 h) treated with NF-CM or NF-exos, CAF-CM or CAF-exos, detected by MTT assay; compared to NF-CM, *p < 0.05, ** *p* < 0.01, ***p < 0.001; compared to NF-exos, # *p* < 0.05, ## *p* < 0.01, ### *p* < 0.001. **c**. Relative IC50 values in cells with different treatment; compared to NF-CM, *p < 0.05, ** *p* < 0.01, ***p < 0.001; compared to NF-exos, # *p* < 0.05, ## *p* < 0.01, ### *p* < 0.001. **d**. Representative images and histogram of cell colony formation of 5637 and T24 cells (pretreated with PTX and DOX for 48 h) (≥ 50 cells were counted as one cell colony under a microscope) treated with NF-CM or NF-exos, CAF-CM or CAF-exos, detected by cell colony formation assay, *** *p* < 0.001. **e**. Representative images and histogram of apoptotic rate of 5637 and T24 cells treated with NF-exos or CAF-exos, detected using flow cytometry, *** *p* < 0.001. **f**. Representative images and histogram of N-cadherin, vimentin and E-cadherin of 5637 and T24 cells treated with NF-exos or CAF-exos, measured by Western blot analysis, *** *p* < 0.001. Data in panels A, B, D, E and F were analyzed using two-way ANOVA, while data in panel C were analyzed using one-way ANOVA, followed by Tukey’s multiple comparisons test. Repetitions = 3. CAF, cancer-associated fibroblasts; NF, normal fibroblasts; CM, conditioned medium; EMT, epithelial-mesenchymal transition; TSG, tumor susceptibility gene; exo, exosome; TEM, transmission electron microscope

First, we set out to obtain the CAFs and NFs from bladder cancer tissues and normal adjacent tissues and to next collect exosomes secreted by these cells. We found that the obtained CAFs and NFs were positive for vimentin and negative for keratin, and that only the CAFs were positive for FAP and α-SMA (Supplementary Fig. 1A). To subsequently investigate the role of CAFs in bladder cancer progression, 5637 and T24 bladder cancer cells were treated with CAF-CM and NF-CM. The cells treated with CAF-CM exhibited a stronger invasive ability (Fig. 1) and drug resistance (Fig. 1) than those treated with NF-CM. TEM was used to assess the morphology of the exosomes (Supplementary Fig. 1B), and nanoparticle tracking analysis revealed that the average size of exosomes was 50–100 nm (Supplementary Fig. 1C). Expression of the exosome markers CD63, CD81 and TSG101 was found to be positive (Supplementary Fig. 1D). The exosomes secreted by the CAFs (CAF-exos) and NFs (NF-exos) were labeled with fluorescent dye PKH67 and added to 5637 or T24 cells to test whether they were internalized. Green fluorescent signals were observed under a laser scanning confocal microscope (Supplementary Fig. 1E), indicating that exosomes labeled by PKH67 were indeed be internalized by the respective bladder cancer cells in a time-dependent manner. No obvious difference was noted in the internalization of CAF-exos and NF-exos (*p* > 0.05). In addition, we found that the CAF-exos markedly increased the migration and invasion of bladder cancer cells compared to the NF-exos (*p* < 0.05; Fig. 1).

By testing the sensitivity of bladder cancer cells to PTX and DOX (pretreated for 48 h) relative to cells treated with NF-CM or NF-exos, we found that the cells treated with CAF-CM or CAF-exos exhibited improved survival and colony formation abilities, as well as lower levels of apoptosis, higher levels of N-cadherin and vimentin expression and lower levels of E-cadherin expression (all *p* < 0.05; Fig. 1), indicating that CAF-exos may induc transition of epithelial cells into mesenchymal cells. These results suggest that exosomes secreted by CAFs may promote the metastasis and chemoresistance of bladder cancer cells by enhancing their epithelial-mesenchymal transition (EMT).

### miR-148b-3p directly enters bladder cancer cells via CAF-exos

**Fig. 2:**
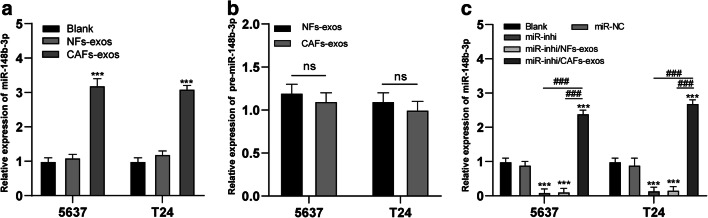
CAF-exos directly transport miR-148b-3p into bladder cancer cells. **a**. Relative expression of miR-148b-3p in 5637 and T24 cells before and after exosome treatment detected using qRT-PCR, *** *p* < 0.001. **b**. Relative expression of pre-miR-148b-3p in 5637 and T24 cells before and after exosome treatment detected using qRT-PCR. **C**. Relative expression of miR-148b-3p in 5637 and T24 cells treated with anti-miR-148b-3p and exosomes detected using qRT-PCR, compared to the blank group, *** *p* < 0.001, in pairwise comparison, ### *p* < 0.001. CAF, cancer associated fibroblast; NF, normal fibroblast; exo, exosome; miR, microRNA. Data in panels A, B and C analyzed using two-way ANOVA, followed by Tukey’s multiple comparisons test. Repetitions = 3

**Fig. 3:**
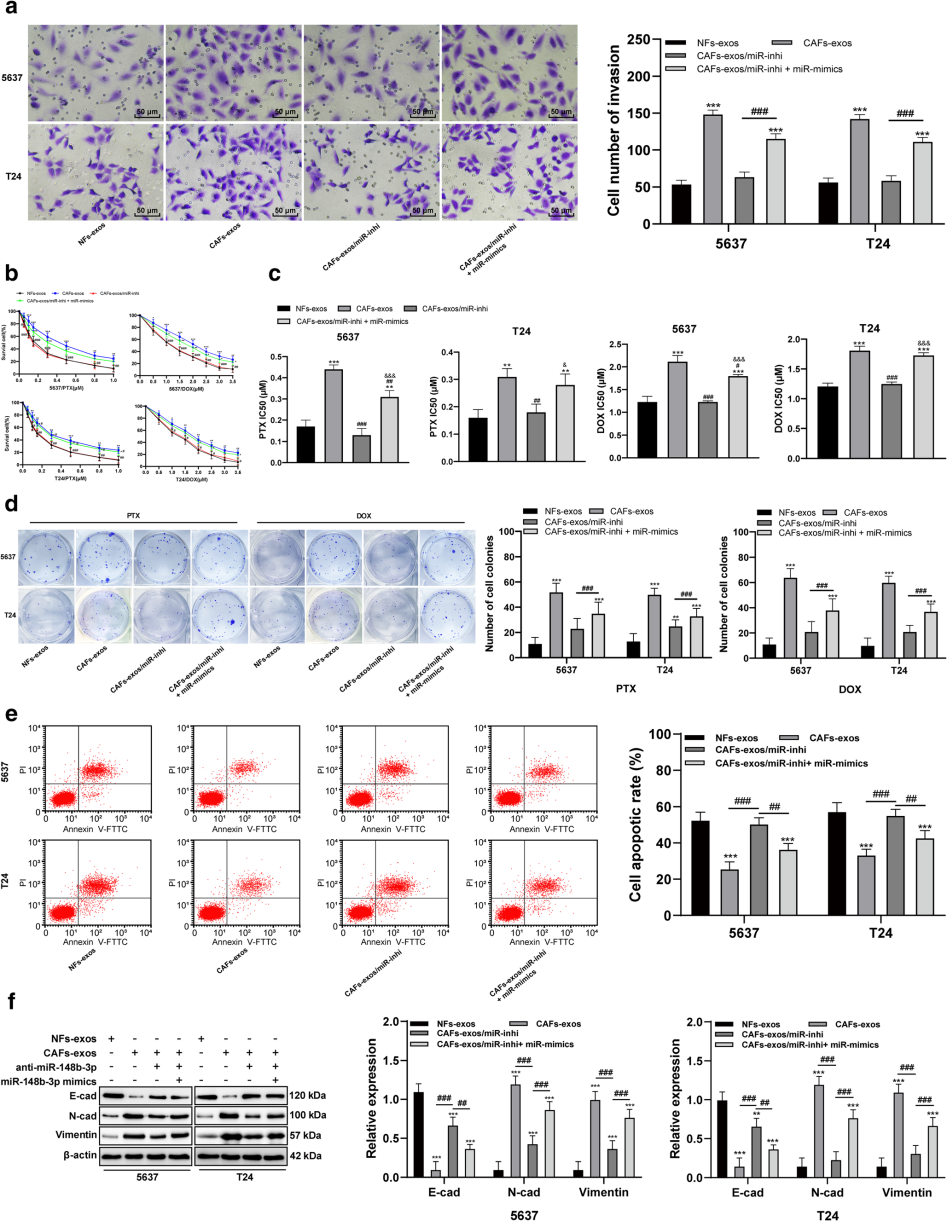
Downregulating miR-148b-3p in CAF-exos inhibits metastasis, EMT and drug resistance in bladder cancer cells. **a**. Representative images and histogram of invaded cells treated with CAF-exos and miR-148b-3p inhibitor detected by Transwell assay, versus cells treated with NF-exos, *** *p* < 0.001, in pairwise comparison, ### *p* < 0.001. **b**. Relative survival rate of 5637 and T24 cells treated with CAF-exos and miR-148b-3p inhibitor measured using MTT assay, versus cells treated with NF-exos, * *p* < 0.05, ** *p* < 0.01, *** *p* < 0.001, versus cells treated with CAF-exos, # *p* < 0.05, ## *p* < 0.01, ### *p* < 0.001. **c**. Relative IC50 values in cells with different treatment, versus cells treated with NF-exos, ** *p* < 0.01, *** *p* < 0.001, versus cells treated with CAF-exos, ## *p* < 0.01, versus cells treated with CAF-exos/miR-inhi, &&& *p* < 0.001. **d**. Representative images and histograms of colony formation of 5637 and T24 cells (pretreatment with PTX and DOX) treated with CAF-exos and miR-148b-3p inhibitor measured with a cell colony formation assay, compared to cells treated with NF-exos, ** *p* < 0.01, *** *p* < 0.001, in pairwise comparison, ### *p* < 0.001. **e**. Representative images and histograms of apoptotic rates of cells treated with CAF-exos and miR-148b-3p inhibitor measured using flow cytometry, relative to cells treated with NF-exos, ** *p* < 0.01,*** *p* < 0.001, versus cells treated with CAF-exos, # *p* < 0.05, ### *p* < 0.001, versus cells treated with CAF-exos/miR-inhi, &&& *p* < 0.001. **f**. Relative levels of EMT-related proteins in cells treated with CAF-exos and miR-148b-3p inhibitor, relative to cells treated with NF-exos, *** *p* < 0.001, in pairwise comparison, ## *p* < 0.01, ### *p* < 0.001. Data in panel C were analyzed using one-way ANOVA, and data in other panels were analyzed using two-way ANOVA, followed by Tukey’s multiple comparisons test. Repetitions = 3. CAF, cancer associated fibroblast; NF, normal fibroblast; exo, exosome; miR, microRNA; EMT, epithelial-mesenchymal transition

The expression of miR-148b-3p is closely related to different types of cancer, and it has been reported that miR-148b-3p levels in sera of bladder cancer patients may be abnormal and, as such, serve as diagnostic markers [12]. We detected the expression level of the 6 miRs in reference samples using qRT-PCR and found that the content of miR-148b-3p in the exosomes was highest (all *p* < 0.05; Supplementary Fig. 2A-B). To verify whether CAF-exos can increase miR-148b-3p levels in bladder cancer cells, we next assessed miR-148b-3p levels in bladder cancer cells treated with CAF-exos or NF-exos, and found the that miR-148b-3p levels in 5637 and T24 cells treated with CAF-exos were substantially enhanced (*p* < 0.05; Fig. 2), suggesting a close association between miR-148b-3p levels and CAF-exos in bladder cancer cells. Additionally, we tested the pre-miR-148b-3p level and found no difference before and after exosome treatment (*p* > 0.05; Fig. 2). Next, we transfected bladder cancer cells with miR-148b-3p inhibitor or miR-NC before incubation with NF-exos or CAF-exos. We found that miR-148b-3p expression was notably reduced in cells transfected with miR-148b-3p inhibitor, but notably elevated in cells incubated with CAF-exos (all *p* < 0.05; Fig. 2). These results indicate that CAF-exos can directly transport miR-148b-3p into bladder cancer cells (Fig. 3).

### PTEN is a target of exosome-mediated miR-148b-3p

As we found that downregulation of miR-148b-3p in CAF-exos can inhibit EMT, metastasis and drug resistance in bladder cancer cells, we next set out to identify miR-148b-3p targets determining these effects in bladder cancer cells. Through online database analysis (http://www.targetscan.org) and screening, we found that miR-148b-3p may target PTEN. The complementary sequence in the PTEN 3’ untranslated region (3’UTR) is shown in Supplementary Fig. 3A. To confirm the targeting relationship between miR-148b-3p and PTEN, we performed a dual luciferase reporter assay and found that miR-148b-3p and PTEN indeed have a targeting relationship. A mutant 3’UTR sequence did not bind to miR-148b-3p, and its fluorescence intensity was significantly higher than that of the WT group (Supplementary Fig. 3B). We also found that the expression of PTEN in bladder cancer cells treated with CAF-exos was lower, which could be reversed by miR-148b-3p inhibitor (all *p* < 0.05; Supplementary Fig. 3C), suggesting that PTEN may serve as a downstream target of miR-148b-3p in exosome-treated bladder cancer cells.

### PTEN upregulation attenuates EMT, metastasis and chemoresistance of bladder cancer cells

**Fig. 4:**
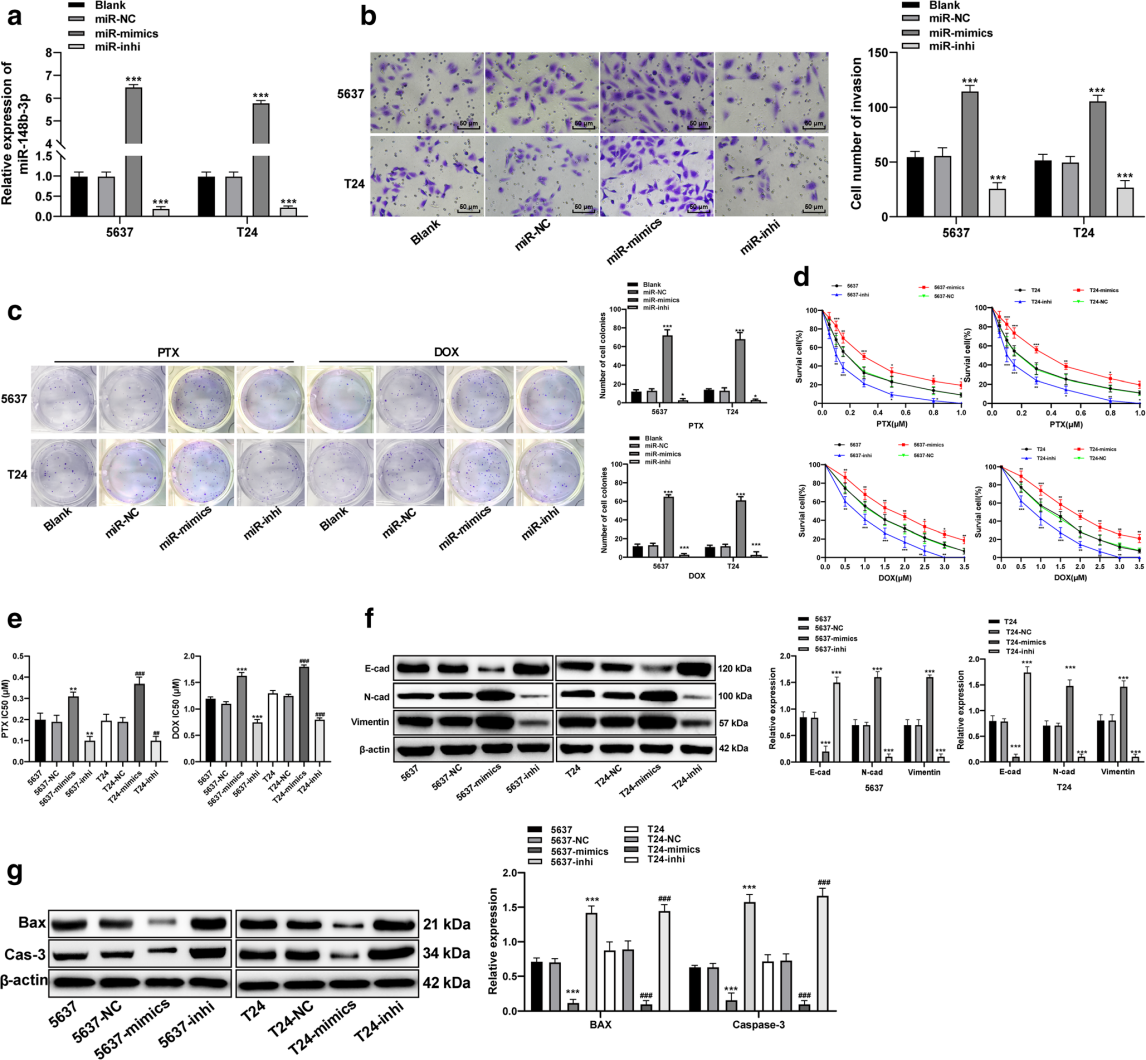
miR-148b-3p upregulation enhances the metastasis, EMT and chemoresistance of bladder cancer cells. **a**. Relative miR-148b-3p expression after transfection of miR-148b-3p mimic or miR-148b-3p inhibitor into 5637 and T24 cells detected using qRT-PCR, compared to the blank group, *** *p* < 0.001. **b**. Representative images and histograms of invaded cells treated with miR-148b-3p mimic or miR-148b-3p inhibitor, compared to the blank group,*** *p* < 0.001. **c**. Representative images and histograms of colony formation of cells (≥ 50 cells were counted as one cell colony under a microscope) treated with miR-148b-3p mimic or miR-148b-3p inhibitor, compared to the blank group,*** *p* < 0.001. **d**. Histograms of viability of cells treated with different concentrations of PTX and DOX for 48 h, compared to 5637 or T24 cells, * *p* < 0.05, ** *p* < 0.01, *** *p* < 0.001. **e**. Relative IC50 values of cells with different treatments; compared to 5637 cells, **p < 0.01, *** *p* < 0.001; compared to T24 cells, ## *p* < 0.01, ### *p* < 0.001. **f**. Relative levels of EMT-related proteins detected using Western blot analysis, relative to 5637 or T24 cells, *** *p* < 0.001. **g**. Relative expression of apoptosis-related proteins detected using Western blot analysis; compared to 5637 cells, *** *p* < 0.001; compared to T24 cells, ### *p* < 0.001. Data in panel F were analyzed using one-way ANOVA, and data in other panels were analyzed using two-way ANOVA, followed by Tukey’s multiple comparisons test. Repetitions = 3. miR, microRNA; EMT, epithelial-mesenchymal transition

After confirming that PTEN may serve as a downstream target of miR-148b-3p, miR-148b-3p mimic or miR-148b-3p inhibitor were transfected into 5637 and T24 cells to up/downregulate miR-138-3p expression (Fig. 4). We found that miR-148b-3p upregulation enhanced bladder cancer cell invasion (Fig. 4. After treatment with chemotherapeutic drugs for 48 h, miR-148b-3p upregulation significantly increased the colony forming capacity and viability of 5637 and T24 cells (Fig. 4), enhanced the levels of N-cadherin and vimentin expression and decreased the level of E-cadherin expression (Fig. 4), indicating that EMT was promoted. Subsequent Western blot analysis revealed that the anti-apoptotic proteins Bax and caspase-3 were significantly downregulated in the bladder cancer cells after miR-148b-3p upregulation (all *p* < 0.05; Fig. 4), indicating repression of apoptosis.

### CAF-exos upregulate miR-148b-3p in mice to promote tumor proliferation and drug resistance

**Fig. 5:**
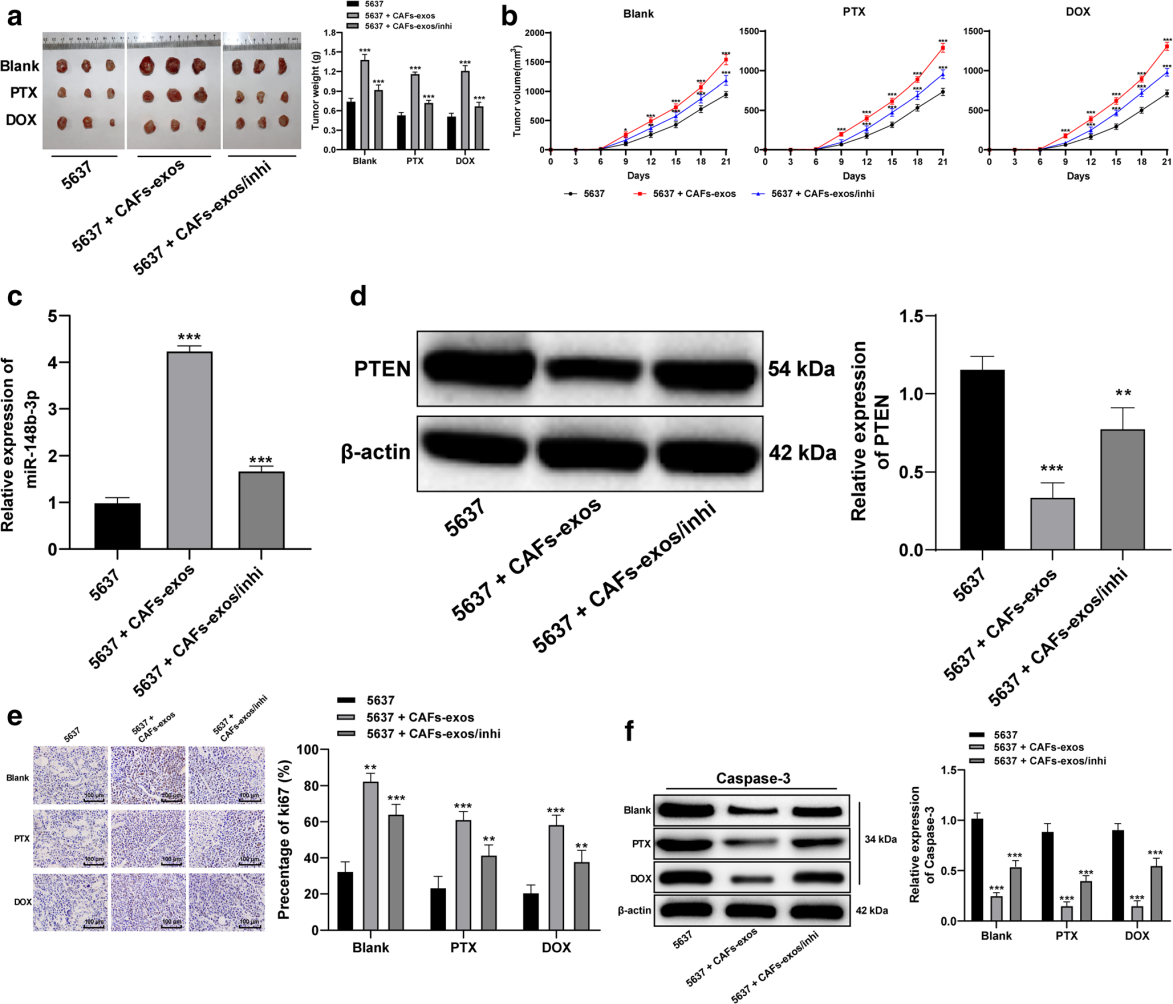
CAF-exos upregulate miR-148b-3p in mice to promote tumor proliferation and drug resistance. **a** and **b**. Representative images and histograms of tumor volume and weight. Tumor volume = (length × width) ^2^/2, compared to 5637 cells, *p < 0.05, **p < 0.01, *** *p* < 0.001. **c** and **d**. Relative levels of miR-148b-3p and PTEN in mouse tumors detected using qRT-PCR and Western blot analysis; compared to 5637 cells, * *p* < 0.05, *** *p* < 0.001. **e**. Representative images of positive Ki-67 rates; compared to 5637 cells, **p < 0.01, *** *p* < 0.001. **f**. Relative caspase 3 expression, compared to 5637 cells, *** *p* < 0.001. 5637 cells (1 × 10^7^/0.2 ml) were subcutaneously injected into each mouse. PTX (10 mg/kg) or DOX (3 mg/kg) was injected into the tail vein every 3 days, and CAF-exos (2–3 µg/mouse) or miR-148b-3p (100 µg/mouse) mixed with transfection reagent (50 µl) were injected into the tail vein according to the grouping. Data in panels C and D were analyzed using one-way ANOVA, and data in other panels were analyzed using two-way ANOVA, followed by Tukey’s multiple comparisons test. n = 06.CAF, cancer associated fibroblast; exo, exosome; miR, microRNA; EMT, epithelial-mesenchymal transition; PTEN, phosphatase and tensin homologue deleted on chromosome 10

For subsequent *in vivo* mouse experiments, 5637 cells were selected. Through subcutaneous injection of these cells in conjunction with PTX, DOX, CAF-exos or miR-148b-3p inhibitor treatment, we found that the volume and weight of the tumors were lowest in the 5637 + miR-inhi group and highest in the 5637 + CAF-exos group (all *p* < 0.05; Fig. 5). In the presence of CAF-exos, the level of miR-148b-3p expression increased, whereas that of PTEN expression decreased significantly in the tumors. In the presence of miR-148b-3p inhibitor the expression of PTEN in the tumors increased significantly (Fig. 5). Through assessment of the Ki-67 proliferation index and caspase-3 expression, we found that compared to the 5637 group, the 5637 + CAF-exos group exhibited a higher Ki-67 index and a lower caspase-3 expression level (Fig. 5). From these results we conclude that miR-148b-3p carried by CAF-exos may enhance the proliferation and drug resistance of bladder cancer cells.

### PTEN reverses the tumorigenic effects of exosomal miR-148b-3p via the Wnt/β-catenin pathway

**Fig. 6:**
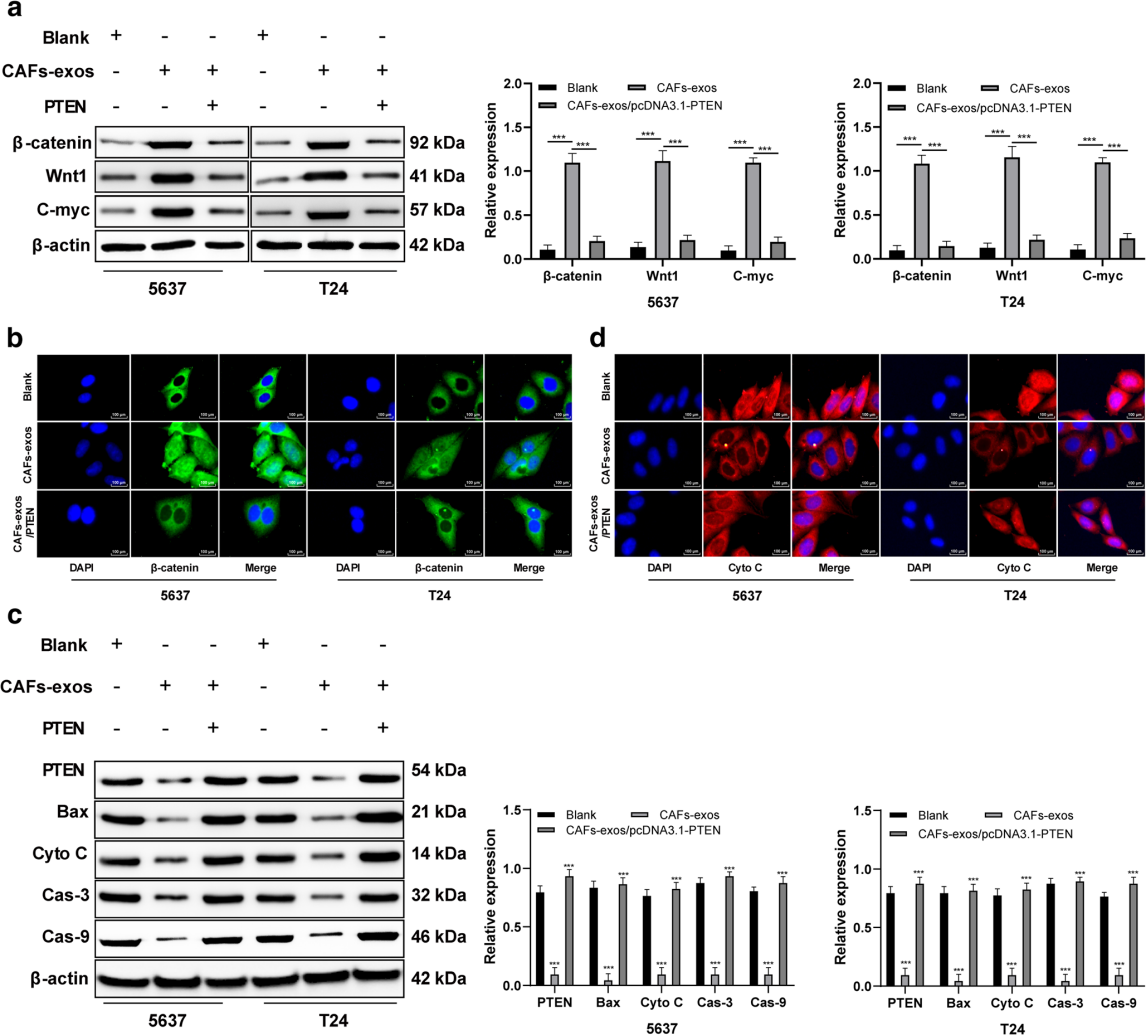
PTEN reverses the tumorigenic effect of exosomal miR-148b-3p via the Wnt/β-catenin pathway. **a**. Relative levels of Wnt/β-catenin pathway-related proteins detected using Western blot analysis, ***p < 0.001. **b**. Representative images of cell morphology after activation of the Wnt/β-catenin pathway. **c**. Relative levels of apoptotic proteins in bladder cancer cells after treatment with chemotherapeutic drugs detected using Western blot analysis,***p < 0.001. **d**. Representative images of cytochrome c distribution before and after PTEN overexpression. Data were analyzed using two-way ANOVA, followed by Tukey’s multiple comparisons test. Repetitions = 3.miR, microRNA; PTEN, phosphatase and tensin homologue deleted on chromosome 10

Recently, it has been found that PTEN may associate with the Wnt/β-catenin pathway in establishing drug resistance [24]. Thus, to reveal potential mechanisms underlying the role of miR-148b-3p in inhibiting the tumorigenic effect of miR-148b-3p on bladder cancer cells, we next set out to study the Wnt/β-catenin pathway. We found that exogenous miR-148b-3p expression significantly upregulated the expression levels of β-catenin, Wnt1 and c-Myc in 5637 and T24 bladder cancer cells, which could subsequently be reversed by PTEN overexpression (Fig. 6). After upregulation of the Wnt/β-catenin pathway the cell morphology changed, which was subsequently restored after PTEN overexpression (Fig. 6). After treatment with chemotherapeutic drugs, exosome-mediated overexpression of miR-148b-3p inhibited the expression of PTEN as well as that of Bax, cytochrome c, caspase-9 and caspase-3. PTEN expression restoration increased the levels of these apoptotic proteins in bladder cancer cells (Fig. 6) and led to cytochrome c release into the cytoplasm (Fig. 6).

## Discussion

Resistance to chemotherapy is a frequent cause of tumor recurrence and treatment failure in bladder cancer [25]. A recent study revealed that inhibition of cancer-derived exosomes may increase the sensitivity of bladder cancer cells to cisplatin [26]. This finding inspired us to test the efficacy of bladder cancer treatment from the perspective of exosomes and drug sensitivity. Using a series of assays we were able to confirm our aforementioned hypothesis that a regulatory loop involving miR-148b-3p, PTEN and the Wnt/β-catenin pathway may be active in bladder cancer cells. From our results we conclude that miR-148b-3p silencing in CAF-exosomes may reinforce bladder cancer cell chemosensitivity through inactivating the Wnt/β-catenin pathway via PTEN upregulation.

First, we found that exosomes secreted by CAFs reduced the apoptosis and promoted the metastasis and chemoresistance of bladder cancer cells by enhancing EMT, indicated by higher levels of N-cadherin and vimentin and lower levels of E-cadherin expression. Others have shown that CAFs can secrete FAP and α-SMA, thereby creating a favorable environment for cancer cells and enabling the migration and invasion of cancer cells [27]. In addition, it has been found that exosomes derived from cancer cells have the potential to expel anticancer drugs and induce chemotherapeutic resistance in malignant cells [28]. It has also been found that bladder cancer cell-derived exosomes can induce cell proliferation and inhibit tumor cell apoptosis *in vitro* in conjunction with upregulated expression levels of Bcl-2, but reduced expression levels of Bax and caspase-3 [29], which is in line with our current observations. Vimentin is a well-recognized metastasis marker [30], while E-cadherin is known to repress the invasion and metastasis of epithelial cells [31]. Additionally, CAF-secreted exosomes have been found to reinforce metastasis and chemoresistance by enhancing EMT in colorectal cancer cells [7]. Based on this information, we propose that inhibition of exosome formation and release may serve as a promising strategy for the treatment of bladder cancer.

Others have shown that abnormal miR-148b-3p levels may be present in sera of bladder cancer patients and, as such, may serve as diagnostic markers [12]. Inspired by this finding, we decided to assess miR-148b-3p expression in CAF-secreted exosomes in order to get insight in its mode of action in bladder cancer. We found that the miR-148b-3p levels were upregulated in bladder cancer cells and CAF-exos and that downregulation of miR-148b-3p in CAF-exos could inhibit EMT, metastasis and drug resistance in bladder cancer cells. It has also been shown that exosomes released from malignant cells may have specific biological activities leading to enhanced proliferation, tumor development, metastasis and resistance to therapy [32]. miR-148b-3p has been shown to strongly correlate with exosome content in paroxysmal nocturnal hemoglobinuria [33]. Similarly, exosomal miR-148a has been found to be overexpressed in the sera of osteosarcoma patients showing a poor chemotherapeutic response [34]. Importantly, it has been found that CAF-secreted exosomal miR-146a may facilitate bladder cancer development [35]. Based on these findings, we speculate that knocking down miR-148b-3p expression in CAF-secreted exosomes may serve as a tool to treat bladder cancer.

We also found that PTEN serves as a target of exosome-mediated miR-148b-3p, and that PTEN upregulation inhibits EMT, metastasis and chemoresistance of bladder cancer cells via the Wnt/β-catenin pathway. PTEN-deficient cells have previously been found to exhibit resistance to rapamycin in advanced transitional cell carcinoma, the most frequently diagnosed type of bladder cancer [36], suggesting a protective role of PTEN in bladder cancer. PTEN deficiency in T24 and HT-1376 bladder cancer cells noticeably attenuated the sh-Jumonji AT-rich interactive domain 2-mediated increase of E-cadherin and decrease of vimentin and N-cadherin expression [37]. Conversely, overexpression of PTEN has been found to inhibit miR-495-mediated proliferation and invasion of bladder cancer cells [38]. Convincing evidence indicates that PTEN loss is associated with urothelial tumor induction, the progression of bladder cancer and its resistance to chemotherapy [39]. A recent study has revealed involvement of PTEN and the Wnt/β-catenin pathway in drug resistance [24], and it has been found that PTEN overexpression can block β-catenin-induced urothelial proliferation and tumorigenesis [18]. In addition, Cullin 4B has been found to promote EMT and metastasis by upregulating the Wnt/β-catenin pathway in bladder cancer [40]. Taken together, we conclude that overexpression of PTEN may reverse the tumorigenic effects of exosomal miR-148b-3p in bladder cancer.

In summary, we found that CAF-exos and exosomal miR-148b-3p can reduce apoptosis and promote EMT, metastasis and drug resistance in bladder cancer cells and that these effects can be reversed by PTEN overexpression via downregulation of the Wnt/β-catenin pathway. These results may be relevant for future (pre-)clinical investigations and applications.

## Electronic supplementary material

ESM 1 Exosome identification. A. Representative images of CAFs and NFs from the bladder cancer tissues and corresponding normal tissues, and the specific proteins identified by immunocytochemistry; B. Representative image of exosome morphology under TEM, showing the average size of exosomes was about 100 nm; C. Exosome size and concentration analyzed by nanoparticle tracking analysis. The maximum peak value is 168 nm, and the concentration is about 2.0 × 106 particles/mL; D. Relative protein bands of exosome markers CD63, CD81 and TSG101 detected using western blot analysis; E. Representative images of exosome internalization labeled by fluorescence and the histogram of relative fluorescence, compared with the 0 hour, *** p < 0.001. Data were analyzed with two-way ANOVA, followed by Tukey's multiple comparisons test. Repetitions = 3. (PNG 1.19 MB)

High Resolution Image (TIFF 10.4 MB)

ESM 2 Relative mRNA expression of 6 miRs in the reference. A and B, Relative mRNA expression of 6 miRs in bladder cancer tissues, normal bladder tissues, 5637 cells, NFs-CM, CAFs-CM, NFs-exos and CAFs-exos detected by RT-qPCR. The 6 miRs are mentioned in the reference (PMID 24961907). Data were analyzed with two-way ANOVA, followed by Tukey's multiple comparisons test. n = 60. Repetitions = 3. (PNG 101 KB)

High Resolution Image (TIFF 914 KB)

ESM 3 PTEN is a downstream target of exosomes-mediated miR-148b-3p. A. The complementary sequence in PTEN 3'UTR through online database analysis (http://www.targetscan.org); B. The targeting relationship between miR-148b-3p and PTEN verified by dual luciferase reporter gene assay. After mutation, PTEN sequence could not be combined with miR-148b-3p sequence, and its fluorescence intensity was significantly higher than that of non-mutation sequence; compared with the NC group, *** p < 0.001; C. Relative expression of PTEN in bladder cancer cells treated with CAFs-exos and miR-148b-3p inhibitor detected using western blot analysis, compared with the blank group, *** p < 0.001. UTR, untranslated region; exo, exosome; miR, microRNA; PTEN, phosphatase and tensin homologue deleted on chromosome 10; NC, negative control. Data were analyzed with two-way ANOVA, followed by Tukey's multiple comparisons test. n = 60. Repetitions = 3. (PNG 214 KB)

High Resolution Image (TIFF 1.61 MB)
