# Supplementary figures and images for: Downregulated exosomal microRNA-148b-3p in cancer associated fibroblasts enhance chemosensitivity of bladder cancer cells by downregulating the Wnt/β-catenin pathway and upregulating PTEN
Source: Cell Oncol (Dordr). 2021 Jan 10;44(1):45–59. doi: 10.1007/s13402-020-00500-0 (PMC7906940; doi:10.1007/s13402-020-00500-0)

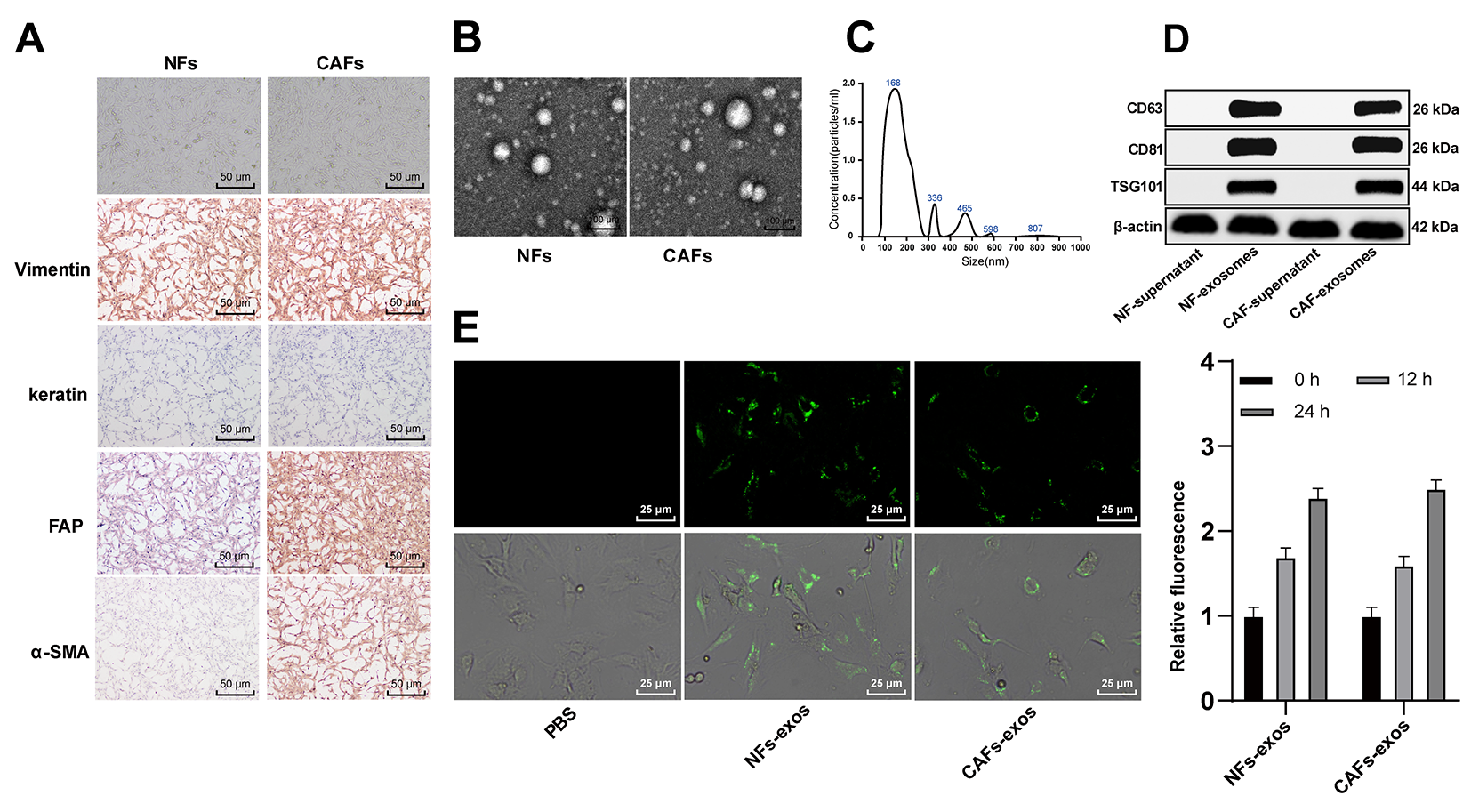

Supplement: Supplementary file 1 — Exosome identification. A. Representative images of CAFs and NFs from the bladder cancer tissues and corresponding normal tissues, and the specific proteins identified by immunocytochemistry; B. Representative image of exosome morphology under TEM, showing the average size of exosomes was about 100 nm; C. Exosome size and concentration analyzed by nanoparticle tracking analysis. The maximum peak value is 168 nm, and the concentration is about 2.0 × 10^6^ particles/mL; D. Relative protein bands of exosome markers CD63, CD81 and TSG101 detected using western blot analysis; E. Representative images of exosome internalization labeled by fluorescence and the histogram of relative fluorescence, compared with the 0 hour, *** p < 0.001. Data were analyzed with two-way ANOVA, followed by Tukey's multiple comparisons test. Repetitions = 3. (PNG 1.19 MB) [file 13402_2020_500_Fig7_ESM.png]

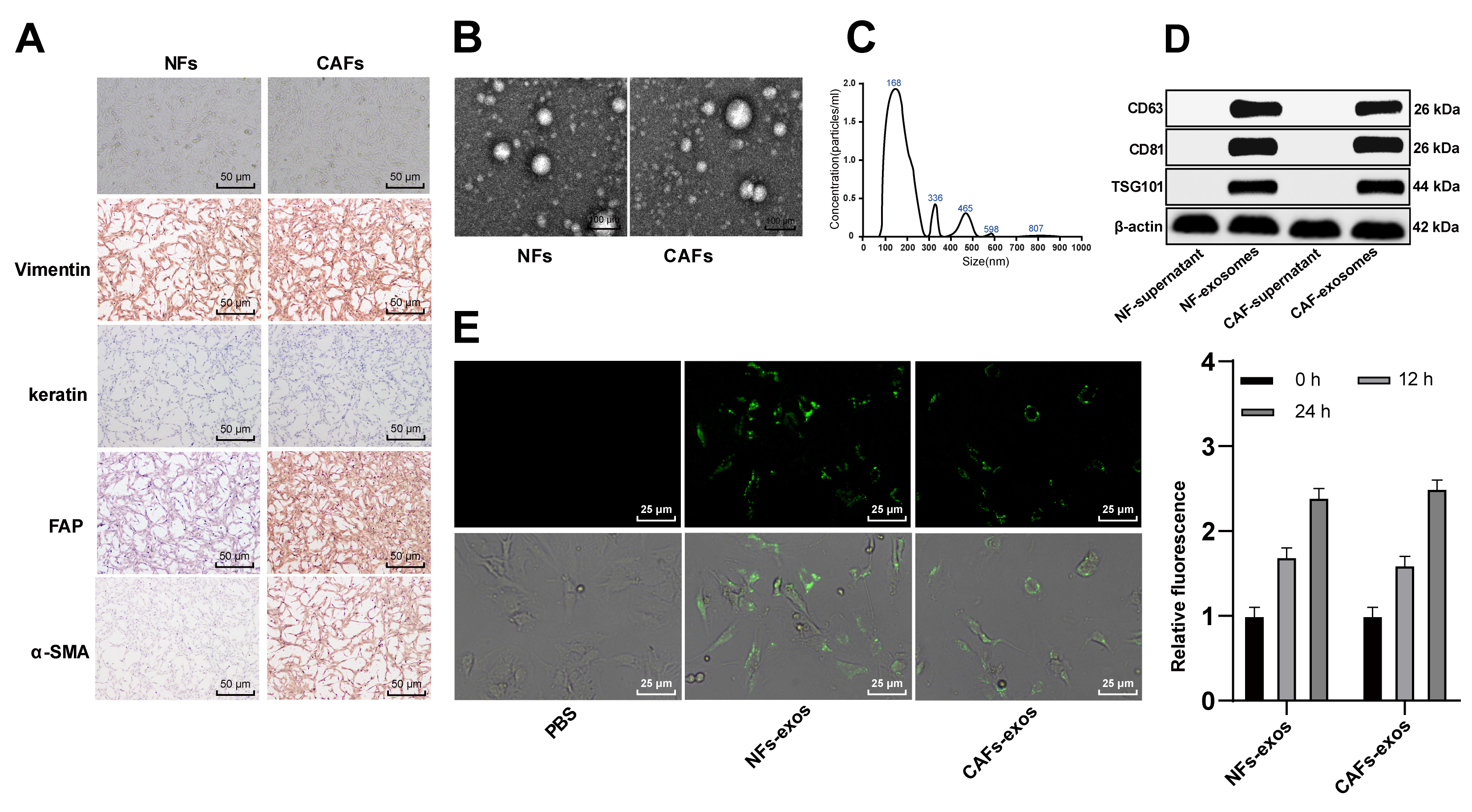

Supplement: Supplementary file 2 — High Resolution Image (TIFF 10.4 MB) [file 13402_2020_500_MOESM1_ESM.tiff]

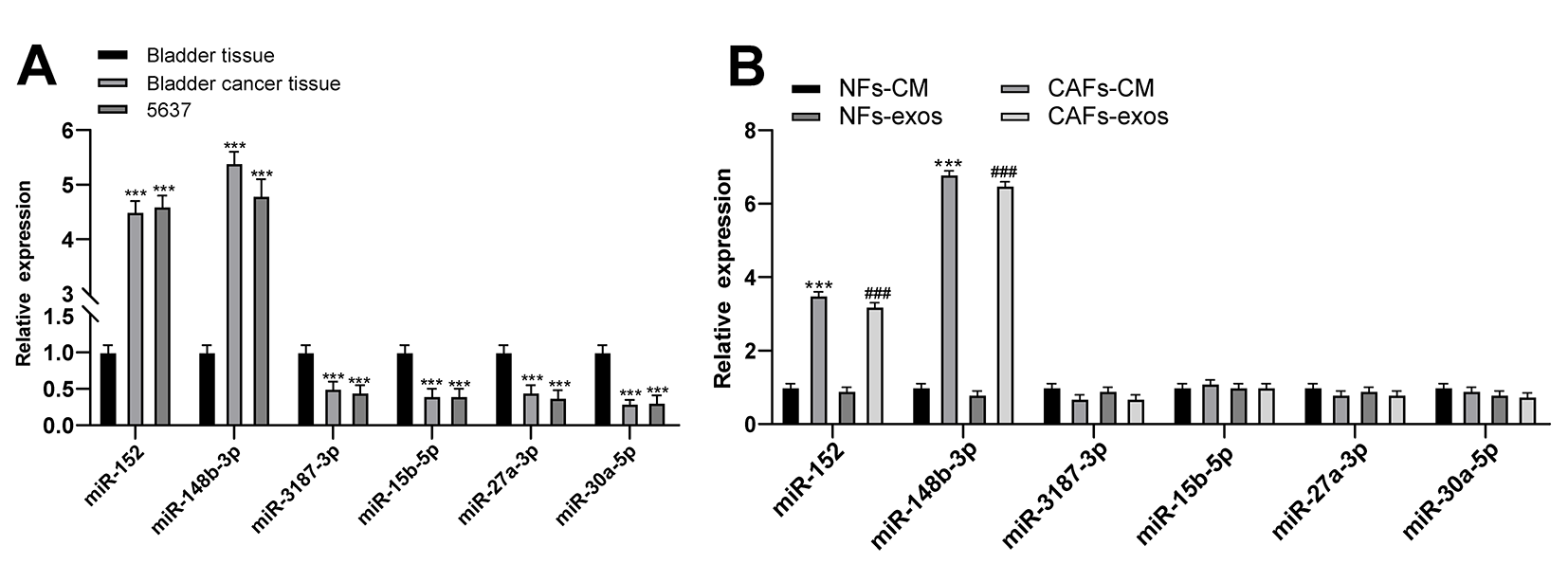

Supplement: Supplementary file 3 — Relative mRNA expression of 6 miRs in the reference. A and B, Relative mRNA expression of 6 miRs in bladder cancer tissues, normal bladder tissues, 5637 cells, NFs-CM, CAFs-CM, NFs-exos and CAFs-exos detected by RT-qPCR. The 6 miRs are mentioned in the reference (PMID 24961907). Data were analyzed with two-way ANOVA, followed by Tukey's multiple comparisons test. n = 60. Repetitions = 3. (PNG 101 KB) [file 13402_2020_500_Fig8_ESM.png]

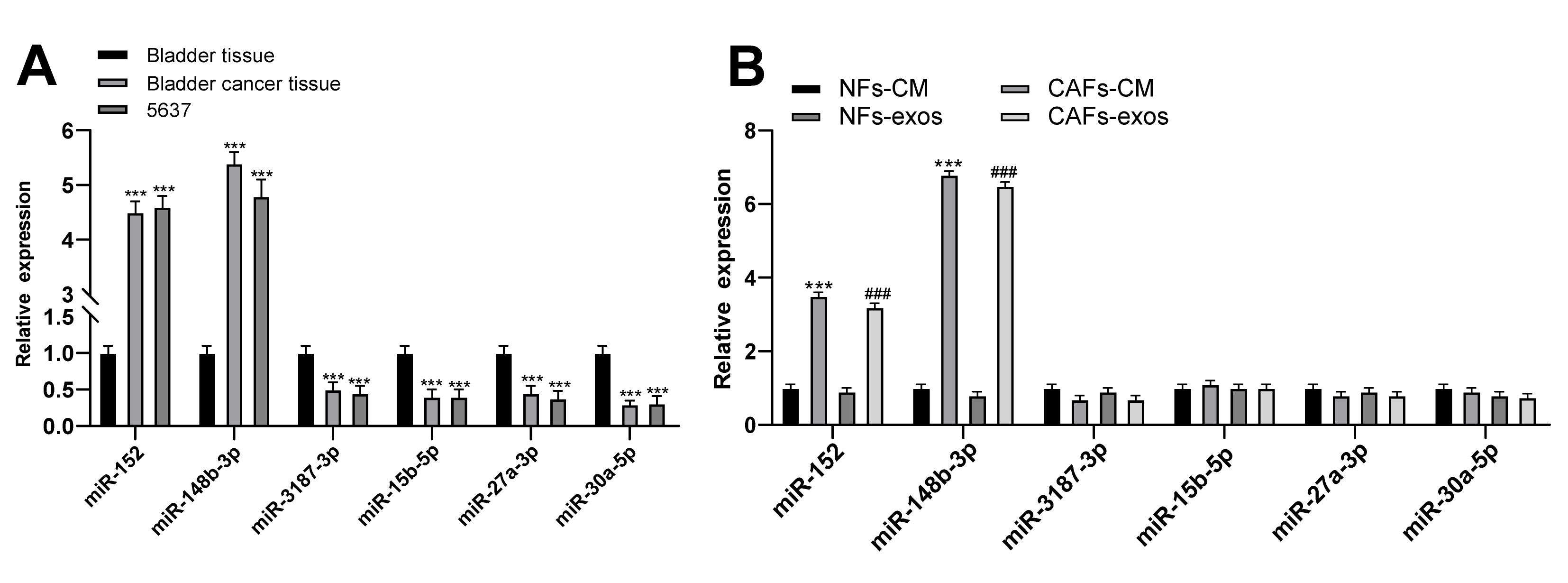

Supplement: Supplementary file 4 — High Resolution Image (TIFF 914 KB) [file 13402_2020_500_MOESM2_ESM.tiff]

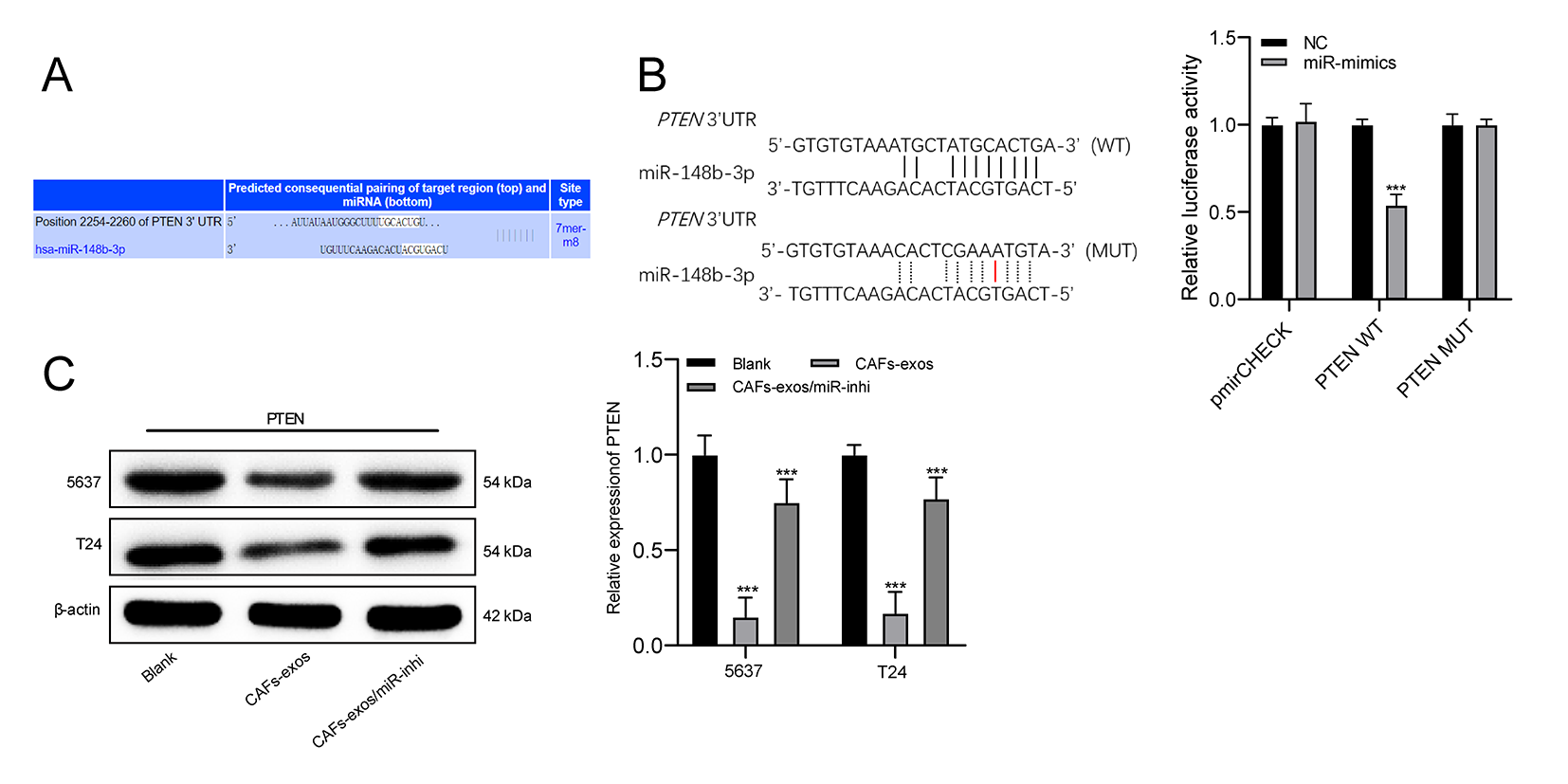

Supplement: Supplementary file 5 — PTEN is a downstream target of exosomes-mediated miR-148b-3p. A. The complementary sequence in PTEN 3'UTR through online database analysis (http://www.targetscan.org); B. The targeting relationship between miR-148b-3p and PTEN verified by dual luciferase reporter gene assay. After mutation, PTEN sequence could not be combined with miR-148b-3p sequence, and its fluorescence intensity was significantly higher than that of non-mutation sequence; compared with the NC group, *** p < 0.001; C. Relative expression of PTEN in bladder cancer cells treated with CAFs-exos and miR-148b-3p inhibitor detected using western blot analysis, compared with the blank group, *** p < 0.001. UTR, untranslated region; exo, exosome; miR, microRNA; PTEN, phosphatase and tensin homologue deleted on chromosome 10; NC, negative control. Data were analyzed with two-way ANOVA, followed by Tukey's multiple comparisons test. n = 60. Repetitions = 3. (PNG 214 KB) [file 13402_2020_500_Fig9_ESM.png]

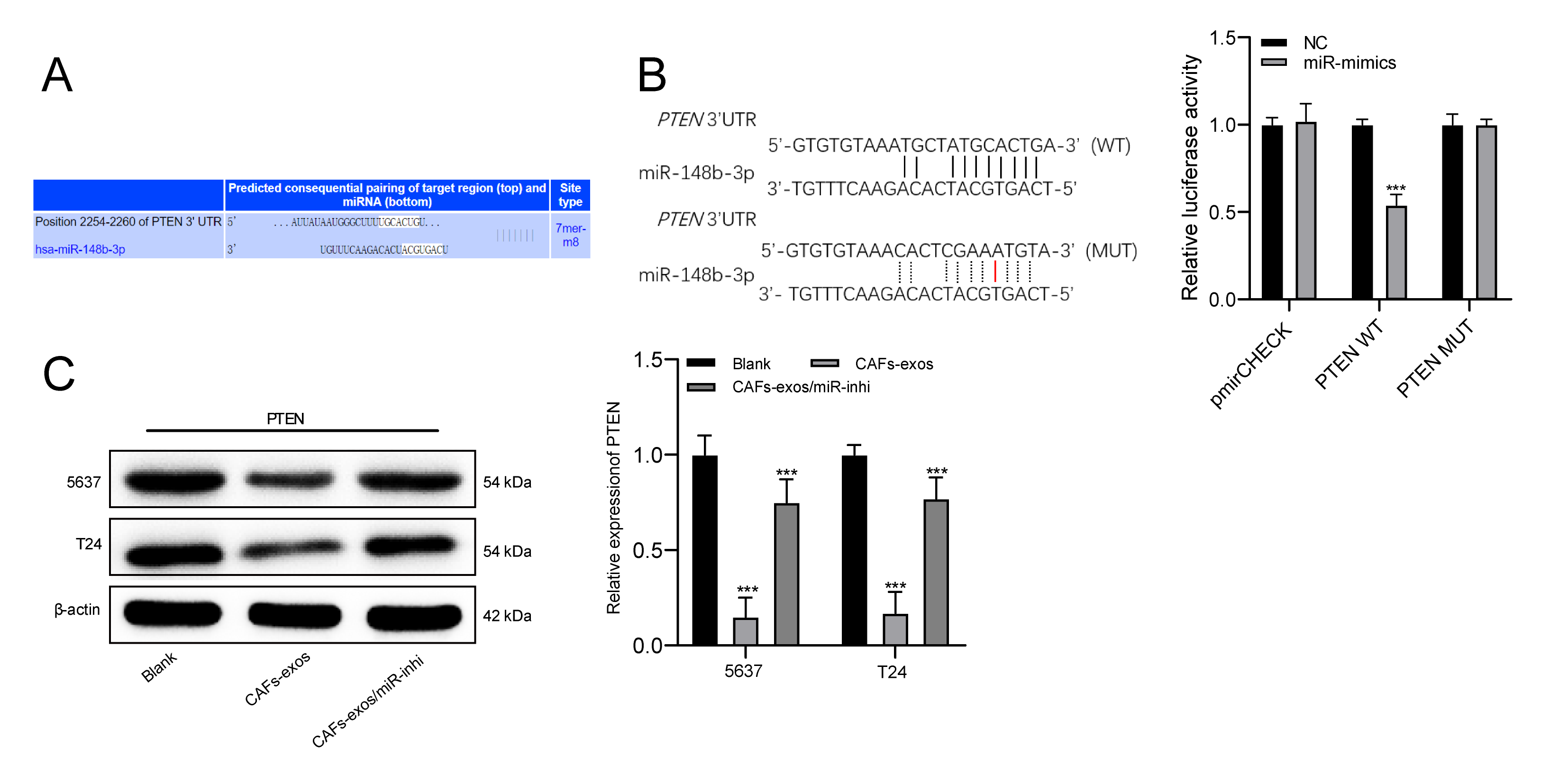

Supplement: Supplementary file 6 — High Resolution Image (TIFF 1.61 MB) [file 13402_2020_500_MOESM3_ESM.tiff]
